# Is protection against florivory consistent with the optimal defense hypothesis?

**DOI:** 10.1186/s12870-016-0719-2

**Published:** 2016-01-28

**Authors:** Adrienne L. Godschalx, Lauren Stady, Benjamin Watzig, Daniel J. Ballhorn

**Affiliations:** Department of Biology, Portland State University, 1719 SW 10th Ave, Portland, OR 97201 USA

**Keywords:** Optimal defense hypothesis, Plant defense, Folivory, Florivory, Cyanogenesis, Lima bean, Direct defense, *Phaseolus lunatus*

## Abstract

**Background:**

Plant defense traits require resources and energy that plants may otherwise use for growth and reproduction. In order to most efficiently protect plant tissues from herbivory, one widely accepted assumption of the optimal defense hypothesis states that plants protect tissues most relevant to fitness. Reproductive organs directly determining plant fitness, including flowers and immature fruit, as well as young, productive leaf tissue thus should be particularly well-defended. To test this hypothesis, we quantified the cyanogenic potential (HCNp)—a direct, chemical defense—systemically expressed in vegetative and reproductive organs in lima bean (*Phaseolus lunatus*), and we tested susceptibility of these organs in bioassays with a generalist insect herbivore, the Large Yellow Underwing (Noctuidae: *Noctua pronuba*). To determine the actual impact of either florivory (herbivory on flowers) or folivory on seed production as a measure of maternal fitness, we removed varying percentages of total flowers or young leaf tissue and quantified developing fruit, seeds, and seed viability.

**Results:**

We found extremely low HCNp in flowers (8.66 ± 2.19 μmol CN^−^ g^−1^ FW in young, white flowers, 6.23 ± 1.25 μmol CN^−^ g^−1^ FW in mature, yellow flowers) and in pods (ranging from 32.05 ± 7.08 to 0.09 ± 0.08 μmol CN^−^ g^−1^ FW in young to mature pods, respectively) whereas young leaves showed high levels of defense (67.35 ± 3.15 μmol CN^−^ g^−1^ FW). Correspondingly, herbivores consumed more flowers than any other tissue, which, when taken alone, appears to contradict the optimal defense hypothesis. However, experimentally removing flowers did not significantly impact fitness, while leaf tissue removal significantly reduced production of viable seeds.

**Conclusions:**

Even though flowers were the least defended and most consumed, our results support the optimal defense hypothesis due to i) the lack of flower removal effects on fitness and ii) the high defense investment in young leaves, which have high consequences for fitness. These data highlight the importance of considering plant defense interactions from multiple angles; interpreting where empirical data fit within any plant defense hypothesis requires understanding the fitness consequences associated with the observed defense pattern.

**Electronic supplementary material:**

The online version of this article (doi:10.1186/s12870-016-0719-2) contains supplementary material, which is available to authorized users.

## Background

Toxic, tough, or unpalatable compounds protect plant tissues against herbivory, making plant defense the gatekeeper mediating food web energy flow. Plant defense patterns vary between plant species and within individuals. To explain this variation, several plant defense theory hypotheses aim to predict the factors driving plant defense patterns [1]. The optimal defense hypothesis (ODH) predicts defense patterns that confer the greatest fitness benefit to the plant and mitigate energetic costs [2]. One cost-saving strategy is differentially protecting organs within the plant, allocating more defense compounds to organs with highest impacts on fitness. Organs predicted to have a particularly high fitness role include reproductive organs as well as active and young vegetative structures that provide the current and future source of photosynthates required for reproduction [1, 3–5]. Testing within-plant defense allocation according to ODH predictions requires understanding 1) the value of each plant part, 2) the benefit of defending that organ, and 3) probability that organ will be attacked [6]. Using these parameters, the aim of this study is to determine whether a plant well-characterized to produce high levels of chemical defense in leaf tissue also invests defensive compounds in flowers, and the role of such pattern according to the ODH.

Plants use many compounds for defense that require amino acids or carbon-based molecules as precursors as well as energy-demanding enzymatic pathways to be produced. Because these precursors would otherwise be used to synthesize proteins or structural compounds, chemical defenses can be costly to the plant [7, 8]. In lima bean (Fabaceae: *Phaseolus lunatus*), one such energetically costly defense, cyanogenesis, requires proteinogenic amino acids and several enzymes to produce cyanogenic precursors (cyanogenic glucosides). For example, the cyanogenic glucosides in lima bean, linamarin and lotaustralin are synthesized from valine and isoleucine [9, 10]. When cells are damaged, two enzymes, β-glucosidase and hydroxynitrile lyase, work sequentially to efficiently release cyanide from the cyanogenic glucosides [11–15]. Taken together, the machinery required to release toxic hydrogen cyanide requires a significant input of nitrogen, which is frequently limited in terrestrial ecosystems. Even legumes, which form a symbiotic relationship with nitrogen-fixing rhizobia face allocation costs due to the photosynthate required to maintain the relationship [16]. Thus, efficiently allocating nitrogen-rich cyanogenic precursors from the source organs to specific and particularly valuable plant tissues would likely lead to higher fitness [15].

Cyanogenesis is an efficient defense against various herbivores, but also incurs costs to the plant in synthesis and transport as well as in ecological interactions [17, 18]. To prevent autotoxicity in the intact plant, vacuolar cyanogenic glucosides are spatially separated from apoplastic β-glucosidases, which combine when herbivores rupture cellular barriers [12]. However, in the absence of herbivores, when faced with plant-plant competition, investment in extensive cyanogenesis can reduce plant fitness [7], reemphasizing the intrinsic costs of this defense. Furthermore, extensive cyanogenesis may make plants more susceptible to fungal pathogens as it has been shown in studies on several cyanogenic plant species such as rubber tree [19] as well as lima bean [20, 21]. To minimize costs, plant cyanogenesis varies among plant organs and in different conditions [16, 22–24]. In lima bean, the experimental plant used in this study, cyanogenic potential (HCNp) depends on various factors. For example, individuals extensively colonized with nitrogen-fixing rhizobia have higher HCNp than conspecifics without the additional source of nitrogen that rhizobia provide [16, 25], and within these plants, young leaves are more cyanogenic. In some plants such as *Eucalyptus cladocalyx*, cyanogenic glucosides are found throughout both vegetative and reproductive structures, and vary temporally resulting from a potential reallocation of cyanogenic resources from leaves to flowering structures [26]. Although lima bean is a well-established model plant in chemical ecology, cyanogenesis of flowers and fruit—organs directly associated with plant fitness—has not yet been measured.

Here we test a key assumption of the ODH: that the within-plant distribution of plant defense reflects the plant organs’ relevance for fitness. To determine quantitative defense investment patterns and resistance to herbivores, we measured cyanogenesis in flower buds, flowers, seed pods as well as in leaves from varying developmental stages, and assessed how much a generalist insect herbivore, the Large Yellow Underwing (Noctuidae: *Noctua pronuba*) would consume each organ. To determine the impact of florivory on plant fitness (defined as number of viable seeds produced per plant) and to compare any impacts with the fitness consequences of folivory (on young, productive leaves), we experimentally removed different percentages of either flowers (0, 25, 50 and 75 %), or young leaf tissue (0, 33, 50 and 66 %). Combining measurements of flower and young leaf HCNp with simulated florivory and folivory experiments enables us to determine the fitness value of each type of organ to the plant and benefit of defending them, while bioassays visualize the probability of flowers and leaves being attacked. If simulated folivory impacts fitness, we expect to see high HCNp in young leaf tissue. If removing flowers significantly reduces plant fitness, we expect flowers and pods will have higher HCNp than vegetative plant tissues, consistent with the ODH. Alternatively, if removing flowers has little or no measurable impact on plant fitness, plants with low cyanogenic flowers and fruit will support the optimal defense hypothesis.

## Results

### Within-plant distribution of chemical defense

**Fig. 1:**
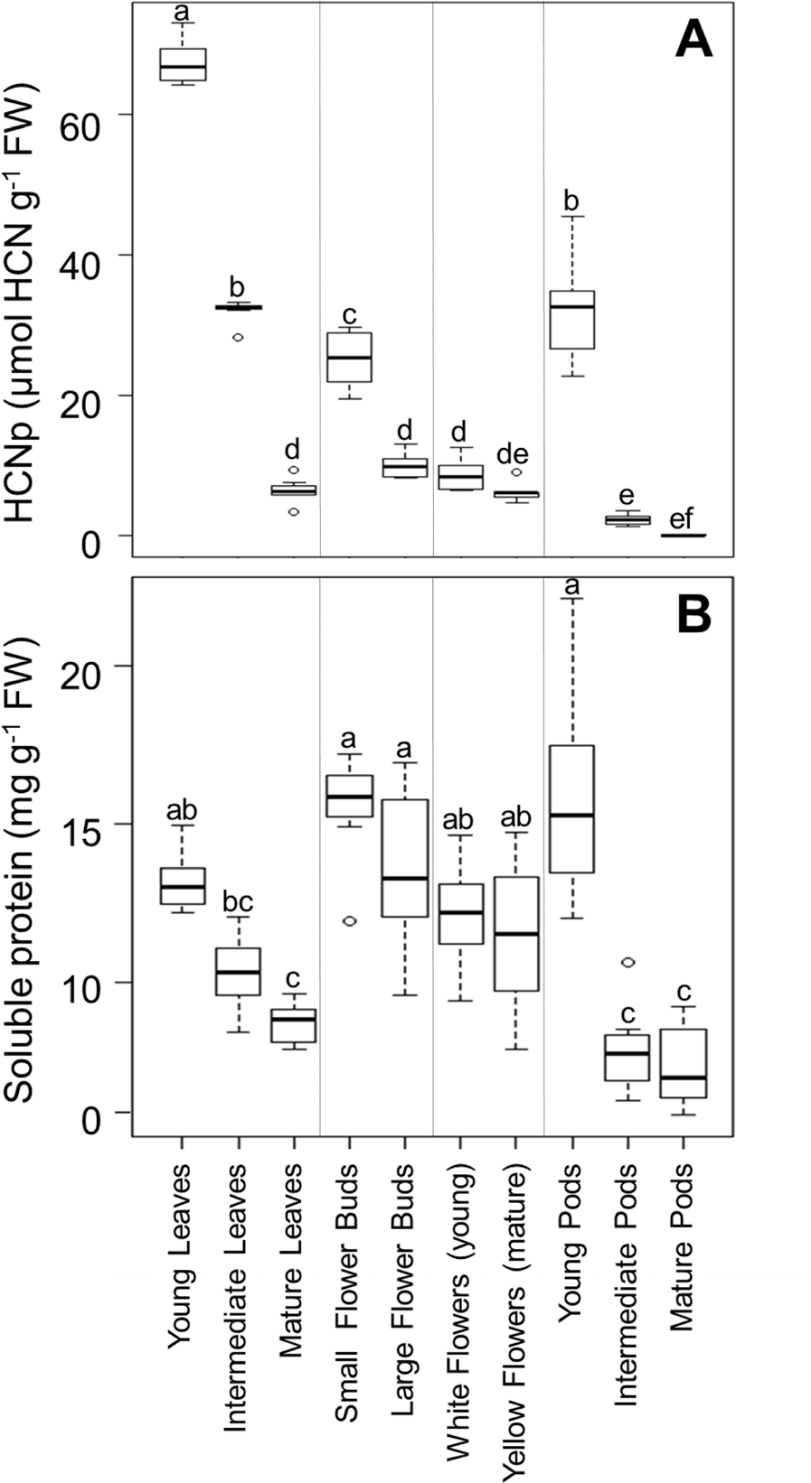
Cyanogenic potential (HCNp; **a**) and soluble protein content (**b**) of different lima bean organs. Boxplots show median plant trait values in bold with rectangles representing the interquartile range from the 1^st^ to the 3^rd^ quartile. Whiskers show minimum and maximum values. Letters indicate significant differences according to posthoc analyses (Tukey’s HSD; *p* <0.05) after one-way ANOVA, *N* = 8

As each organ matured (flower buds, flowers, pods, and leaves), the cyanogenic potential (HCNp) for that organ decreased. The reproductive organs with the highest HCNp were young pods with 32.05 ± 7.08 μmol CN^−^ g^−1^ FW, which decreased to almost non-detectable levels of 0.09 ± 0.08 μmol CN^−^ g^−1^ FW as pods developed to intermediate and mature pods, making mature pods that are preparing for senescence the lowest cyanogenic plant organs [Fig. 1a, one-way ANOVA: F_1,9_ = 381.64, *p* <0.001; Tukey’s HSD, *p* <0.05]. These low levels of cyanide are also found in the more mature developmental stages of flowers. In small flower buds, HCNp is the second highest among reproductive organs, which decreased as flower buds grew larger, and further decreased when flowers first bloomed (white petals), and then matured and changed color to yellow. Yellow flower HCNp is not significantly different from the lowest cyanogenic organs (intermediate and mature pods) with 6.23 ± 1.25 μmol CN^−^ g^−1^ FW (Fig. 1a). By contrast, young leaves contained the highest concentration of cyanide with an average HCNp of 67.35 ± 3.15 μmol CN^−^ g^−1^ FW (Fig. 1a). As leaves developed into intermediate and mature stages, HCNp significantly decreased relative to the highly cyanogenic young leaves (Fig. 1a, one way ANOVA: F_1,9_ = 381.64, *p* <0.001). Intermediate leaves had similar HCNp values as young pods, and mature leaves had HCNp values not significantly different from the lower cyanogenic flower buds and flowers.

### Soluble protein concentration

Similar to HCNp, soluble protein content (an important nutritive trait: [27]) in lima bean organs decreased with maturity (Fig. 1b). Between organs, protein content differed significantly (F_1,9_ = 21.68, *p* <0.001) with young leaves, small flower buds, large flower buds, flowers, and young pods all containing higher protein concentrations than mature leaves, and both intermediate and mature pods (Fig. 1b). We found no significant difference in total soluble protein content between young leaves, the most cyanogenic organ, and all flower developmental stages, one of the least cyanogenic organs (Fig. 1b). Thus, flowers have the highest nutritive value: low defense, but high protein.

### Cafeteria-style feeding trials

**Fig. 2:**
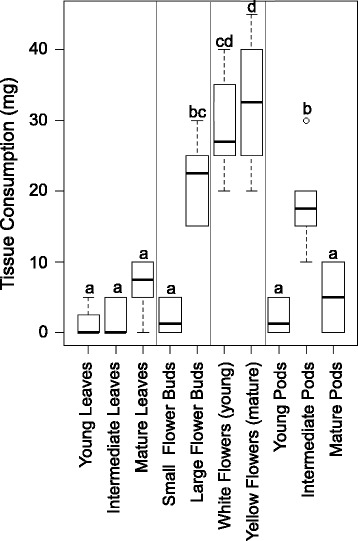
Tissue consumed by generalist herbivores. Different plant organs were offered to *Noctua pronuba* larvae in choice feeding trials and tissue consumption was determined. Boxplots show median tissue consumption by in bold with rectangles representing the interquartile range from the 1^st^ to the 3^rd^ quartile. Whiskers show minimum and maximum values. Letters indicate significant differences according to posthoc analyses (Tukey’s HSD; *p* <0.05) after one-way ANOVA, *N* = 6 feeding trials

Variation in HCNp among organs resulted in significant differences in fresh weight of food consumed, showing variation in herbivore food choices (one-way ANOVA, F_1,9_ = 31.369, *p* <0.001). Insects preferred organs with the lowest HCNp, with the exception of mature pods and leaves (Fig. 2). Among the low cyanogenic tissues, herbivores preferred flowers more than any other tissue, followed by large flower buds, and intermediate pods, both of which released <20 μmol CN^−^ g^−1^ FW. HCNp decreased as pods developed, but the mature stage pods also began to develop tougher, mechanically defended tissue in preparation for senescence. Both young and mature pods were consumed significantly less than intermediate pods (Fig. 2). Compared with any leaf tissue, herbivores in this experiment consumed three times more flower tissues (Fig. 2).

### Plant fitness consequences of florivory

**Fig. 3:**
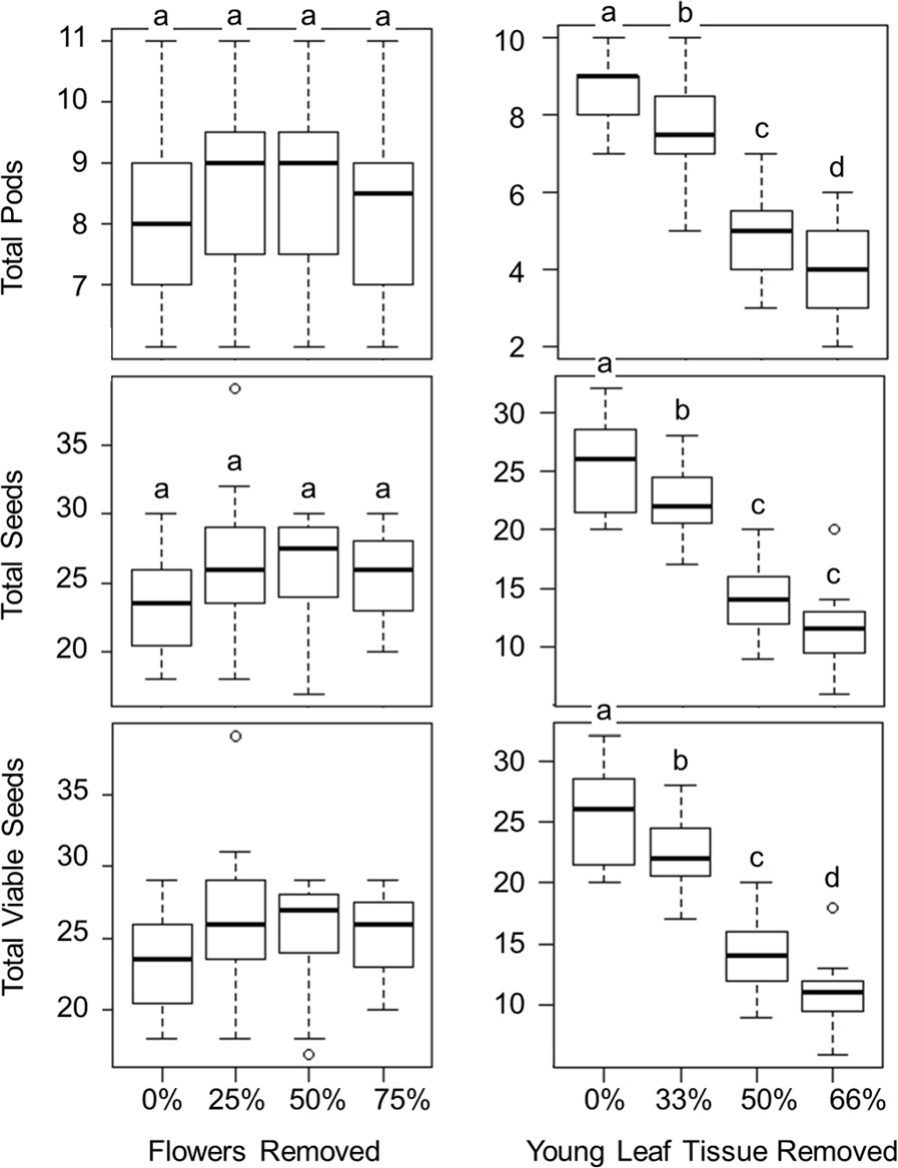
Pod and seed production following simulated florivory or folivory. Pod and seed production as well as the number of viable seeds of lima bean plants with different percentages of either flower or young leaf tissue removal were quantified. Tests for differences between flower removal treatments from one-way ANOVAs: total pods, *p* = 0.707, total seeds, *p* = 0.189, and viable seeds, *p* = 0.108, *N* = 20. Tests for differences between young leaf tissue removal treatments from one-way ANOVAs: total pods, *p* <0.001, total seeds, *p* <0.001, and viable seeds, *p* <0.001, *N* = 20. Boxplots show median values in bold with rectangles representing the interquartile range from the 1^st^ to the 3^rd^ quartile. Whiskers show minimum and maximum values. Letters indicate significant differences according to posthoc analyses (Tukey’s HSD; *p* <0.05)

Simulating florivory by removing flowers had no measurable impact on plant maternal fitness. The reproductive output per plant, measured as the number of viable seeds, was not affected by simulated florivory treatments (Fig. 3). Removing 0, 25, 50 %, or 75 % of flowers did not significantly affect pod number (one-way ANOVA, F_1,3_ = 0.466 *p* = 0.707), total seeds (F_1,3_ = 1.634, *p* = 0.189), or total viable seeds (F_1,3_ = 2.098, *p* = 0.108).

### Plant fitness consequences of young leaf folivory

Simulating folivory on young leaves did significantly decrease plant maternal fitness. The reproductive output per plant, measured as the number of viable seeds, quantitatively decreased as leaf removal was experimentally increased (Fig. 3). Removing 0, 33, 50 %, or 66 % of young leaf tissue reduced final pod number (one-way ANOVA, F_1,3_ = 80.475 *p* <0.001), total seeds (F_1,3_ = 77.530, *p* <0.001), and total viable seeds (F_1,3_ = 94.261, *p* <0.001).

## Discussion

In this study we tested one prediction of the optimal defense hypothesis (ODH), which states plants should allocate defense compounds towards tissues that are most relevant for plant fitness [1]. Testing such investment in defense traits across different plant organs requires all organs to rely on the same kind of defense [28]. We show here that lima bean plants accumulate the defensive compounds, cyanogenic glucosides, in all aboveground plant tissues tested [14, 18]. Comparing the cyanogenic potential (HCNp) of flower buds, flowers, pods, and leaves from several developmental stages, we found that the organs with the highest HCNp were not the reproductive organs (i.e., flower buds, flowers, and pods), which directly determine plant fitness, but instead were young leaves. These findings are consistent with cyanogenic patterns in *Eucalyptus cladocalyx* in which young leaves have the highest HCNp among all organs [26]. In our study, we conducted feeding trials with generalist insect herbivores to assess the probability that herbivores would attack each organ and found that—corresponding to their low HCNp and high nutritive value—the insects preferred flowers among all tested plant organs. In nature, lima bean is attacked by various generalist and specialist herbivores [29, 30]. We frequently observed noctuid generalist caterpillars feeding during the night on various organs of lima bean plants including all organs tested in this study as well as generalist locusts feeding on the same tissues during the day. Thus, larvae of the generalist noctuid moth species (*N. pronuba*) selected for this study seem suitable for bioassays with lima bean tissues.

According to the ODH, attack risk is one factor that should increase defense compound allocation to that organ, and in this case flowers seem to have a high risk for attack but low defense, counter to theoretical predictions [1, 2, 31]. To experimentally quantify the benefit to the plant associated with defending flowers, we compared the flowers’ low HCNp to the fitness value of that organ in simulated florivory experiments. The fitness consequences associated with florivory revealed support for the optimal defense hypothesis because removing flowers does not impact our metric for measurable fitness: viable seed production. Thus, in our system, the number of individual flowers does not critically determine the reproductive output per plant individual.

If removing a portion of total flowers does not reduce seed production, investing resources towards defense compounds in flowers likely does not maximize fitness. In fact, compared to plants with all flowers intact, in our study removing any percent of flowers causes a slight, but not significant, *increase* in number of pods, total seeds, and viable seeds. This phenomenon has been described in another study on *Solanum carolinense*, where flower removal designed to simulate weevil damage stimulates mature fruit production [32]. In *Phaseolus vulgaris*, a plant species closely related to our experimental plant, removing flowers between day 11 and 20 within the flowering period can increase seed yield [33]. Because flowers contribute differentially to final seed yield depending on timing within the flowering period [34], we removed a given percentage of flowers continuously throughout the flowering period to exclude any flowering timing effects. Overall, the lack of reduced seed number and viability with varying degrees of simulated florivory helps to explain the low concentrations of cyanogenic glucosides in flowers. In fact, if some florivory stimulates seed production, this could potentially select against highly cyanogenic flowers. Whether slight florivory is favored or whether cyanogenic glucoside costs outweigh the benefits of defending flowers, the distribution of cyanogenic glucosides we observed suggests that lima bean plants allocate chemical defense to young leaves rather than to reproductive tissues.

High concentrations of cyanogenic glucosides in young leaves as we observed suggests that plants allocate these compounds from the source organs—likely intermediate, fully photosynthetically active leaves (Ballhorn, unpubl. data)—to the young leaf sinks. Young leaves likely have an important fitness contribution due to their role as future producers of photosynthates important for growth and reproduction. In this line, plants with various levels of simulated folivory produced fewer pods, seeds, and viable seeds in this study in a quantitative damage-response relationship. High protection of young, expanding leaves is a consistent pattern with other studies that test the optimal defense hypothesis [4, 5, 35–37]. Herbivores attack young leaves >20 times more often than more mature leaves [38]. Given the risk for attack and value as a potential future carbon source organ, plants often protect young leaves relatively more than mature leaves [39–41]. Young leaves of *Eucalyptus cladocalyx* consistently have the highest concentrations of cyanogenic precursors [24, 26]. Terpenoid concentrations are highest in the young leaves of *Solidago altissima*, which impact capitula mass more than other tissue when removed [5]. Barto and Cipollini [3] removed leaves from various developmental stages of *Arabidopsis thaliana* and also concluded young leaves can be the most valuable plant organ for measurable plant fitness. Our HCNp data in concert with our data showing fitness consequences of removing young leaf area are consistent with the optimal defense hypothesis with fitness-relevant organs, young leaves in this case, being the most cyanogenic.

### How do multiple defenses interact to shape organ-specific levels of chemical defense?

In addition to having the highest cyanogenic potential in our study, young lima bean leaves produce the highest quantities of extrafloral nectar and volatile organic compounds, both of which are indirect plant defenses, attracting enemies of the plant’s herbivores to protect the plant [16, 36]. Frequently plants employ multiple defense strategies, including indirect defenses to protect against their diversity of attackers [42–44]. Among these defenses, tradeoffs between direct and indirect or inducible defenses can be adaptive to conserve resources and maximize fitness, consistent with the optimal defense hypothesis [1, 28, 45]. Several traits in lima bean trade off with cyanogenesis, including several mechanical, inducible, and indirect defenses [43]. Lima bean genotypes with consistently high cyanogenic potential produce less extrafloral nectar, carbon-based volatile organic compounds, and are more susceptible to pathogen attack [20, 43, 46]. The sum of plant defense interactions against attack on all plant parts, including both florivory and folivory, may help explain the distribution of any individual defense compound within plant tissues. For example, phenolic glycosides concentrations in *Populus tremuloides* leaves were 30 % higher when leaves also contained extrafloral nectaries [47], which follows the pattern of extrafloral nectar secretion and cyanogenesis in lima bean. This pattern could be consistent with optimal defense predictions to protect against different feeding guilds, or if investing resources towards one defense makes that organ important to protect. For example, leaves that secrete extrafloral nectar can serve as a significant resource sink [48], and plants may have higher fitness by protecting the carbon investment. However, this dual protection pattern contradicts the optimal defense hypothesis if investing in multiple defenses is redundant, or if the plant’s defenses deter or harm beneficial insects. An example of this occurs in *Mentzelia pumila* plants, which have trichomes that trap and kill predatory coccinellid beetles [49]. Trichome density as a mechanical defense covaries with cyanogenesis, with hook-shaped trichomes expressed in greater frequency in highly cyanogenic lima bean genotypes, putatively as a mechanism to protect tissues against a broader range of herbivores with different feeding strategies [43]. Chewing herbivores effectively rupture cellular barriers between enzymes and precursors, but herbivores such as phloem- or cell content feeders that can avoid extensive damage may be more affected by barriers to accessibility, including hook-shaped trichomes, or even tissue toughness. In our study, the decreasing HCNp fruits and leaves which goes hand in hand with simultaneously increasing toughness of these organs may further indicate an ontogenetically shift from chemical to mechanical defense. The plant’s interacting defense traits and resulting within-plant distribution may be constrained by the network of ecological interactions, both with herbivores from various feeding guilds or with beneficial mutualists that can contribute to plant fitness.

### Ecological implications of florivory and folivory

Plant defense distribution throughout various organs likely also depends on the ecological value of protecting those organs. Some interspecific interactions greatly impact plant fitness, such as plant-pollinator or plant-microbe interactions, which may be significantly compromised by either folivory or florivory. Symbiotic, nitrogen-fixing rhizobia in legume root nodules can consume up to 20–30 % of the plants’ total photosynthate pool [50], and intense leaf area removal by folivores can starve other plant organs of carbon when photosynthesis is limited [51]. Quantitative leaf removal also reduces extrafloral nectar secretion, a reward plants use to attract natural predators such as ants [48]. Leaf removal, herbivory, and simulated herbivory alter flower size and shape, which not only impacts the energy reserves within the reproductive structure, but also may attract fewer pollinators [52–54]. Pollen per plant and pollen quality can decrease with leaf consumption [55]. Flower consumption can reduce the strength of the visual or chemical signals that attract pollinators [56]. Reduced pollination decreases male fitness by limiting pollen transfer [57]. By altering rates of outcrossing, florivores can act as a selection pressure for entire mating systems, increasing the frequency of selfing, which can have severe fitness consequences [58]. Therefore, although we were not able to measure the fitness consequences associated with ecological interactions, protecting various organs with high levels of chemical defense may be partly explained in the context of plants maintaining mutualistic interactions.

## Conclusions

In our study, we test the optimal defense hypothesis (ODH) by assessing the cyanogenic potential of reproductive and vegetative organs in a highly cyanogenic plant. By comparing floral cyanogenesis, the risk that generalist herbivores would consume floral tissue, and the fitness value of flowers, we examined the factors expected to affect organ-specific defense: 1) value of organ, 2) benefit of defense, and 3) probability for attack [6]. Within-plant distribution of cyanogenic potential—low in flowers, but highest in young leaves—reflects the fitness relevance of the reproductive and vegetative organs, and is consistent with the within-plant assumption of the optimal defense hypothesis. Our HCNp data show that measuring plant traits in various organs alone does not provide a comprehensive picture of defense resource allocation, but combining plant defense patterns with bioassays and evaluating fitness is a more powerful approach to determine whether or not the observed patterns align with any theoretical framework for plant defense. The optimal defense hypothesis continues to be a leading hypothesis because the underlying premise enables many plant trait patterns to maximize fitness in the right context. Although this is not the first call to action requiring a big-picture perspective of how plant defenses interact to shape defense allocation patterns, our data emphasize the role of fitness benefits and consequences shaping plant defense distribution patterns. As we continue to measure empirical patterns in plant defense allocation, it becomes increasingly apparent that the fitness consequences and ecological context are both essential for understanding how, when, and where plants protect themselves.

## Methods

### Plant cultivation

Lima bean plants (genotype CV 8078, [20]) were cultivated in a greenhouse adjusted to resemble conditions at natural lima bean habitats in Costa Rica (30 °C/24 °C, 75–85 % humidity, 14 h/10 h light/dark photoperiod). Lights in the greenhouse were a combination (1:1) of HQI-BT 400 W (Osram) and RNP-T/LR 400 W (Radium) lamps with a photon flux density of 550–700 mol photons m^−2^ s^−1^ at table height. Plants were cultivated in plastic pots of 15 cm in diameter in a 1:1 ratio of potting soil (Fox Farms, Arcata, CA) and sand (grain size 0.5 mm). Plants were watered daily and fertilized with 50 ml of a 0.1 % aqueous solution of Flory-3 fertilizer [NPK plus magnesium (%); 15, 10, 15, +2; EUFLOR, Munich, Germany] weekly. To simulate resource allocation patterns that more closely resemble organ tissue development within natural populations, plants were inoculated with 10 mL liquid culture of a lab-maintained rhizobia strain isolated from wild lima bean plants in Costa Rica. Position of plants in the greenhouse was rotated every 3 days to exclude position effects. Feeding experiments and analyses of plant chemical traits were conducted after a cultivation period of 8 weeks.

### Insect rearing

Caterpillars of the Large Yellow Underwing (*Noctua pronuba*) were used in the feeding trials. This insect species represents an extremely polyphagous herbivore feeding on a broad range of herbaceous and woody plants. Caterpillars were reared from eggs in July 2012 and were fed with non-cyanogenic raspberry leaves to avoid adaptations to cyanide-containing food. *Noctua pronuba* is an invasive pest insect in the United States. Eggs were collected on private property in Raleigh Hills (DJ Ballhorn, Portland, OR). Neither field work nor collection of caterpillar eggs required permits. Our research is in compliance with all relevant guidelines and/or appropriate permissions.

### Cyanogenic potential (HCNp)

The cyanogenic potential (HCNp; total amount of cyanide present accumulated in a given tissue) was quantified for leaves, flower buds, flowers and fruit from different developmental stages. For preparation of plant extracts, fresh samples weighed to the nearest 0.001 g were ground with liquid nitrogen in a pre-cooled (4 °C) mortar and pestle. Plant material was homogenized in 3 mL ice-cold aqueous Na_2_HPO_4_ solution (67 mmol L^−1^). Enzymatic hydrolysis of cyanogenic precursors was conducted with specific β-glucosidase isolated from rubber tree (Euphorbiaceae: *Hevea brasiliensis*), a plant containing the same cyanogenic glucosides (linamarin and lotaustralin) as lima bean. We used enzyme solution adjusted to an activity of 20 nkat. Samples were incubated for 20 min at 30 °C in a water bath in closed glass vessels (Thunberg vessels) [18, 59] and the HCNp was quantified by enzymatic hydrolysis of cyanogenic precursors and subsequent spectrophotometric detection of released cyanide at 585 nm using the Spectroquant® cyanide test (Merck, Darmstadt, Germany).

### Soluble protein content

Concentration of soluble protein in flower, fruit and leaf samples was quantified according to Bradford [60] with modifications following Ballhorn et al. [17, 60]. Bradford reagent (Biorad Laboratories, Munich, Germany) was diluted 1:5 with ddH_2_O and 20 μL of each homogenized plant sample was combined with 1 mL of diluted Bradford solution. Bovine serum albumin (BSA; Fluka ChemieAG, Buchs, Switzerland) at different concentrations was used to create a standard curve. After 5 min incubation time, concentration of protein was spectrophotometrically measured at 595 nm. We used the same individual plant extracts for protein measurements as for HCNp analyses, thus, both parameters were quantitatively attributed to the same sample.

### Feeding trials

Cafeteria-style feeding trials were conducted in Petri dishes (9 cm; *N* = 6 feeding trials) lined with moist filter paper to avoid water loss of samples. Each dish contained one insect herbivore (3rd larval stage). Pre-weighed plant leaf samples (leaf discs, 1 cm in diameter), flower buds, flowers and fruits (large fruits were presented in form of discs cut out with a cork borer; 1 cm in diameter) were offered simultaneously to the insects over a time period of 2 h. Plant fresh material consumption was determined by re-weighing the plant samples. A control set of each organ was weighed and reweighed after 2 h to control for potential evaporation and change in mass due to non-consumptive effects. As we did not observe detectable weight loss for any of the fresh plant samples in this control we did not consider spontaneous evaporation as a factor potentially affecting our results.

### Flower removal effects on fitness

To assess fitness consequences of florivory, a given percentage of flowers were mechanically removed throughout the experiment from each plant to create four treatments: 0 % flowers removed, 25 % flowers removed, 50 % flowers removed, and 75 % flowers removed. Flowers were removed at a medium flower bud stage and treatments were repeated every 3 days throughout the flowering period. The experimental duration covered the whole period from the formation of the first inflorescence to the opening of the last flowers. After seeds matured fully, the pod and seed production for each plant was counted and seed viability per plant was determined by germinating seeds on moist paper towels until a healthy radicle developed.

### Young leaf removal effects on fitness

**Fig. 4:**
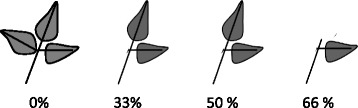
Simulated folivory treatment experimental design. Four treatment groups with different percentages of young leaf tissue removal were established as depicted

To assess fitness consequences of folivory, developing young trifoliate leaves were mechanically damaged to create four treatments: 0, 33, 50 and 66 % leaf area removal (Fig. 4). These percentages were modified from the ones utilized in the flower removal experiments based on the nature of the trifoliate leaf (Fig. 4). Initial leaf area removal was applied when plants developed their first fully unfolded secondary leaf, and experimental leaf tissue removal was continuously applied (once per week) to newly developed, but unfolded leaves throughout the experimental period. Maternal fitness including pod number, seed number and viability were measured as in flower removal experiments.

### Statistical analyses

Data for HCNp, protein content, cafeteria experiment feeding trials, and flower removal experiments were all analyzed using One-Way ANOVA tests followed by Tukey’s post hoc tests in R Studio [61].

## Availability of data

Data are found in supplementary files. All data have been provided (See Additional files 1, 2, and 3).

## Additional files

Additional file 1:
**Plant Trait and Bioassay Data.** Organ indicates the plant tissue tested, Protein indicates mg protein g^−1^ FW, and HCNp indicates the cyanogenic potential as μmol CN^−^ g^−1^ FW. Bioassay results appear in the green box below plant chemical trait data. (XLSX 26 kb) Additional file 2:
**Simulated Florivory Data.** # of Flowers indicates the total number of flowers, and for each of the flower removal treatments (25, 50 and 75 %) the total number of flowers removed follows each respective column. The total pods, seeds, and viable seeds are indicated below #fruit, #seeds, and #viable seeds. (XLSX 14 kb) Additional file 3:
**Simulated Folivory Data.** Treatment indicates the percentage of young leaf tissue removed from the plant; leaves indicates the total leaves per plant prior to removal; fruit, seeds, and viable indicate total pods, seeds, and viable seeds at the end of the experiment. (XLSX 11 kb)

## Abbreviations

HCNp: cyanogenic potential
ODH: optimal defense hypothesis
